# Effect of the Addition of Different Vegetal Mixtures on the Nutritional, Functional, and Sensorial Properties of Snacks Based on Pseudocereals

**DOI:** 10.3390/foods10102271

**Published:** 2021-09-26

**Authors:** Ana Karen González-Calderón, Natalia Alejandra García-Flores, Ana Sofía Elizondo-Rodríguez, Mariana Zavala-López, Silverio García-Lara, Néstor Ponce-García, Anayansi Escalante-Aburto

**Affiliations:** 1 Department of Nutrition, Universidad de Monterrey, Av. Ignacio Morones Prieto 4500 Pte, San Pedro Garza García 66238, Mexico; ana.gonzalezc@udem.edu (A.K.G.-C.); natalia.garciaf@udem.edu (N.A.G.-F.); ana.elizondor@udem.edu (A.S.E.-R.); 2 Tecnologico de Monterrey, Escuela de Ingeniería y Ciencias, Ave. Eugenio Garza Sada 2501, Monterrey 64849, Mexico; mariana.zavala@itesm.mx (M.Z.-L.); sgarcialara@itesm.mx (S.G.-L.); 3 Faculty of Agricultural Sciences, UAEMex, Campus Universitario ‘El Cerrillo’, El Cerrillo, Piedras Blancas s/n, Toluca 50200, Mexico

**Keywords:** vegetal proteins, phenolic compounds, antioxidant activity, sensorial attributes, ancient grains

## Abstract

Quick meals available in markets are popular among consumers. Generally, these products are not recognized as functional foods owing to nutrient-poor composition. In this study, energy snack bars were developed with different formulations, using puffed quinoa, amaranth, cacao liquor, and coconut oil, and the effects of the addition of commercial vegetal mixtures (VM) on nutritional and functional properties were assessed. VM addition showed significant effects on the protein, lipid, and fiber contents, phenolic compounds (PHC) content, and antioxidant activity of the snacks. The control snack showed higher levels of free and bound PHC. The oxygen radical absorbance capacity (ORAC) analyses recorded highest values of free PHC (9392.7 μmol TE/100 g dry weight) in PC65 (concentrate based on a combination of vegetal proteins), whereas the highest bound PHC levels of 47,087 and 46,531 μmol TE/100 g dry weight were observed in PC65 and the control snacks, respectively. Sensorial attributes assessment provided a high score on the hedonic scale, wherein panelists detected no differences among the samples. Altogether, the selection of non-conventional ingredients with high antioxidant activities emerged as a successful strategy to produce sensory acceptable meals.

## 1. Introduction

In the past few years, snacks, with or without substantial nutritional value, have emerged as an alternative to quick meals. In fact, snacks are quite popular among consumers of all ages. Generally, most of these products are not recognized as functional foods, mainly due to their nutrient-poor composition. In the recent past, there has been a growing interest in the manufacturing of new types of snack bars using functional components. Therefore, such snack bars can also be included under the category of functional products, while keeping in mind consumers’ acceptability and suitability of the product as ready to eat [1]. In general, functional foods are defined as dietary items that provide nutrients and energy, and at the same time positively modulate one or more targeted functions in the body, by enhancing certain physiological responses and/or reducing the risk of certain diseases [2].

Consumption of ancient foods has gained significant attention as these foods introduce different nutrient sources into the actual human diet, and thus assist in counteracting the high ingestion of ultra-processed foods [3]. The seed of quinoa, *Chenopodium quinoa* Wild, is a pseudocereal, which has been under cultivation for more than 5000–7000 years, in the Andean region and other South American countries. Since prehistoric age, quinoa has been used as a substitute cereal for consumption [4]. Quinoa seeds are endowed with exceptional nutritional and functional properties. This grain contains ~14.8 g/100 g of dietary fiber and ~16.5 g/100 g of proteins (with 20 amino acids, 10 of which are essential). Additionally, it is quite rich in unsaturated fatty acids, including linoleic acid (C18:2, ω-6) and α-linolenic acid (C18:3, ω-3) [5,6]. Functionally, quinoa seeds are gluten-free, and contain ~103.6 mg of gallic acid equivalents (GAE)/100 g of dry weight (d.w.) of total polyphenols [7]. Interestingly, the high amounts of unsaturated fatty acids (66.9–76.53%), total carotenoids index (496.1–738 μg/g d.w.), and total tocopherols index (9.3–93.6 μg/g d.w.) present in quinoa seeds has been previously shown to demonstrate a good correlation with antioxidant activity, evaluated using FRAP, oxygen radical absorbance capacity (ORAC), and DPPH assays [6,8].

Amaranth (*Amaranthus* ssp.) is a pseudo-cereal, which has been popularly consumed since the pre-Colombian age. Several previous studies have reported the presence of high nutritional value proteins and high content of lysine in amaranth [9,10]. Importantly, amaranth is characterized by total phenol content of 15.5 mg GAE/100 g d.w., anthocyanin content of 83 mg of cyanidin 3 glucoside/100 g d.w., and flavonoids content of 70.2 mg catechin eq/100 g d.w. [11]. Cacao liquor (*Theobroma cacao*) is a rich source of both monounsaturated and saturated fatty acids. Oleic acid is one of the most important components of cacao liquor. Palmitic and stearic acids are the primary saturated fatty acids present in cacao. Additionally, cacao contains some minerals, including magnesium, copper, potassium, and iron. Among the polyphenolic compounds, catechins, anthocyanins, and proanthocyanidins are the most abundant class of compounds present in cocoa powder [12].

Substitution of animal proteins by vegetal proteins still remains controversial; however, a significant growth has been recorded in the market for vegan and vegetarian consumers. The 2015 Dietary Guidelines Advisory Committee maintained primary focus on higher dietary patterns of plant-based foods in their scientific report [13]. It was recommended that the empty calories contributed by added sugars should be replaced, in part, with a better variety of plant protein. Thus, the general perception related to plant protein has evolved over the past few years [14]. Initially, plant proteins were perceived as less nutritious and an incomplete source of essential amino acids, but now they are considered as a healthy alternative to fulfill protein needs and recommendations [15]. All the aforementioned ingredients are endowed with exceptional nutritional and functional properties, as reported in previous studies. In order to fulfill the sustainable objectives of the Food and Agricultural Organization [16], it is required to improve the nutritional and functional values of food products in the future.

Several commercially available snack bars are added with vegetal mixtures; however, just a few are assessed by scientific methods. The effect of the vegetal mixtures containing different compounds such as proteins in the nutritional and texture properties of snacks have been investigated by Malecki et al. [17]. Algae, pumpkin, sunflower, rice, soy, and hemp, were used as ingredients to produce high-protein bars. However, glucose syrup (~32% *w*/*w*) was added, considered a non-desirable component for a healthy diet.

Microbiological and sensory properties of diet bars elaborated with chia grain (*Salvia hispanica* L.) and soy protein were evaluated by Veggi et al. [18]. They concluded that the addition of chia seeds has a positive effect on the sensory characteristics of this type of product.

There are no scientific reports of the antioxidant assessment of snack bars containing only vegetal mixtures to our knowledge. Therefore, the present study aimed to evaluate the effect of the addition of different vegetal mixtures (VM) on the nutritional properties and phenolic contents of snacks prepared using non-conventional ingredients, with minimal processing. Additionally, the antioxidant activity and sensorial attributes of different snack samples were evaluated. 

## 2. Materials and Methods

### 2.1. Materials

Commercially available puffed quinoa, amaranth, cacao liquor, coconut oil, and coconut milk were used for the development of different formulations. In order to obtain different ingredient combinations and produce the energy snacks, three VM were used, namely Provita C^®^ (Nutrigrains^®^, Monterrey, Mexico), an isolate based on a combination of vegetal proteins that contained 88% proteins, 7% carbohydrates, and 2% lipids, d.w.; Provita C65^®^, a concentrate based on a combination of vegetal proteins that comprised of 60% proteins, 38% carbohydrates, and 2% lipids, d.w.; and Probalance^®^ (Nutrigrains^®^, Monterrey, Mexico), a high dietary fiber ingredient that is based on the combination of a vegetal protein, containing 20% proteins, 40% carbohydrates, 35% dietary fiber, and 3% lipids, d.w. [19].

### 2.2. Energy Snacks Production

**Figure 1:**
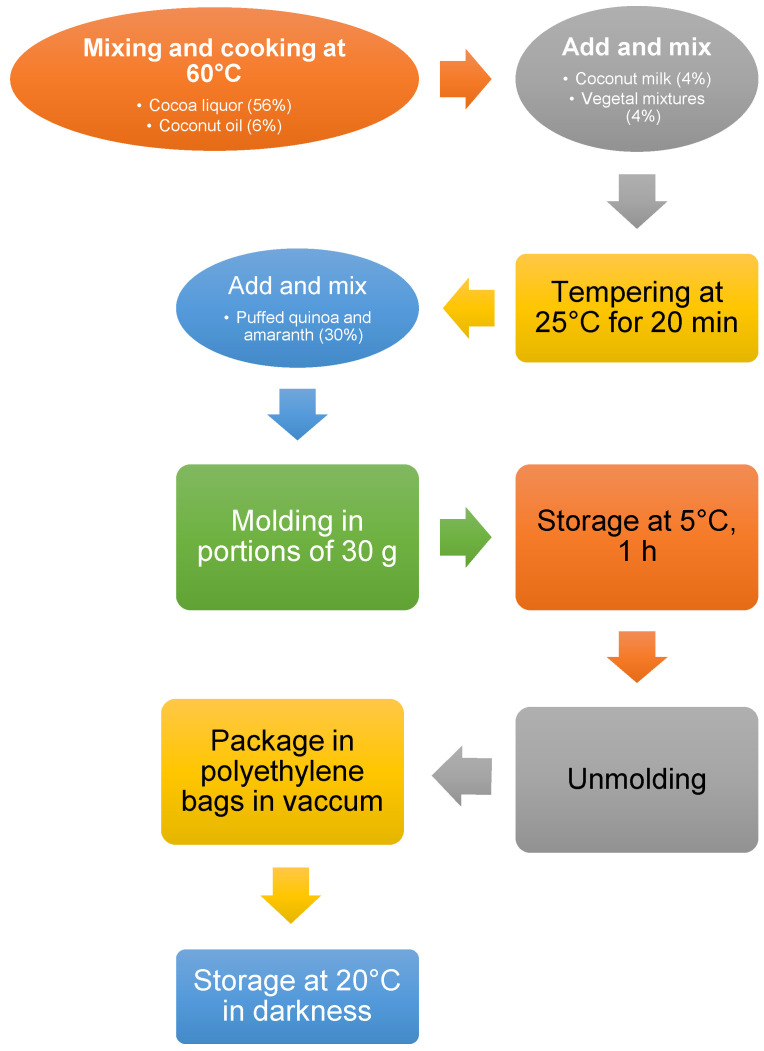
General methodology followed for the development of energy snacks with vegetable mixtures.

**Figure 2:**
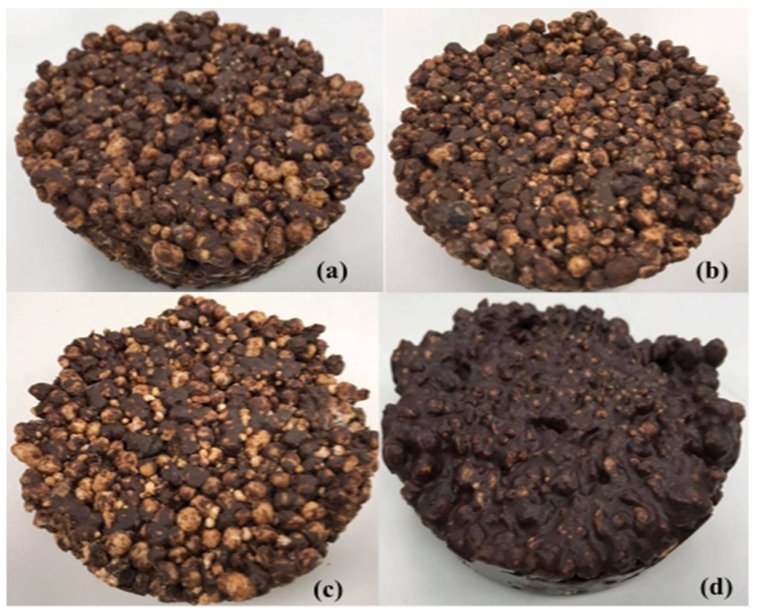
Samples of different energy snacks produced using different vegetable proteins mixtures. (**a**) PC, (**b**) PC65, (**c**) PB, and (**d**) control (without protein mixture).

Several preliminary assays were performed to obtain the final formulation for a product with optimum sensorial characteristics, similar to the products commercially offered in the market. The snacks were prepared at the Gastronomy Laboratory of the University of Monterrey. The production of the bar samples was carried out as per the criteria of NOM-251-SSA1-2009 [20]. General methodology followed for the production of the energy snacks is shown in Figure 1. For confidentiality reasons, the proportion of the ingredients used in the production has not been reported in detail (patent application under process). The products were developed using four treatments: PC: Base formula + Provita C^®^; PC65: Base formula + Provita C65^®^; PB: Base formula + Probalance^®^; and Control: Base formula without vegetal mixture (VM). The final products obtained after the elaboration of the energy snacks is shown in Figure 2, in particular, the products containing VM presented similar characteristics, except for the control snack.

### 2.3. Nutritional Analysis

Sample products obtained from each treatment were ground into a fine powder using a blender (Moulinex, model 980-18, France), and passed through a sieve (60 US). The resulting samples were transferred to polyethylene bags, sealed, and stored in dark at 5 °C in a refrigerator until used further. All nutritional analyses were conducted using the methods approved by AACCI [21]. The moisture content was calculated using AACC method 44-01.01. For the assessment of fat acidity, AACC method 02-01.02 was used, while ash content or inorganic material was measured using AACC method 08-01.01. The protein content was evaluated using AACC method 46-10.01. Total sugars and reduced sugars were quantified according to the procedure previously described by Eynon and Lane [22]. Total dietary fiber was assessed using a commercially available assay Kit (Sigma Aldrich, St. Louis, MO, USA), which was based on the 958.29 AOAC method. It combined enzymatic and gravimetric parameter for the determination of fiber content. All assays were performed in triplicates. Total calories by portion (30 g of product) were calculated using Equation (1), as per the specifications described by Kraisid et al. [23].

(1) Calories per portion (kcal/30 g)=[(g of carbohydrates+g of protein) 4+(g of lipids) 9]

### 2.4. Determination of Phenolic Acids (PHC, Bound and Free)

#### 2.4.1. Extraction of PHC

A microscale method was used to assess bound and free PHC present in the samples, according to the procedure previously described by Zavala-López and García-Lara [24]. To extract soluble (free) PHC, 50 mg of defatted, dehydrated, and homogenized sample of each bar was mixed with 0.7 mL of 80% methanol. Further, the samples were incubated at 25 °C for 2 h with continuous stirring at 450 rpm. After sedimentation for 15 min, the supernatant was carefully removed and the sampled was incubated at room temperature for 24 h to ensure complete solvent evaporation. After 24 h, 0.7 mL of solvent (80% methanol) was added to the residue pellet, and the sample was vortexed at 2500 rpm for 5 min. The bound PHC were obtained from the soluble fraction of the residue pellet. Briefly, 2 M NaOH was added to the pellet and volume was reduced from 10 mL to 0.5 mL. Alkaline hydrolysis was performed at 90 °C, with constant agitation at 500 rpm. Following this, acidification (pH 2) was achieved by the addition of 0.5 mL of 2 M HCl. To remove lipid, 0.8 mL of n-hexane was added to the sample, which was vortexed at 2500 rpm for 5 min. The upper hexane layer was discarded, and the washing procedure using n-hexane was repeated twice. Following this, the bound PHC were recovered using ethyl acetate. Briefly, 0.8 mL ethyl acetate was added, and the sample was vortexed at 2500 rpm for 5 min. Further, the sample was incubated at 25 °C, and aforementioned steps of constant agitation and centrifugation were performed. Next, ethyl acetate was evaporated, and the resulting dry residue was re-suspended in 200 µL of 50% methanol. Finally, the suspension was filtered through a 0.45 µm GHP membrane and a Nylon filter. Both the extracts for soluble and bound PHC were stored at −20 °C until used for further analysis.

#### 2.4.2. Quantification of Free and Bound PHC

The amount of free and bound PHC were quantified according to the procedure described by Zavala-López and García-Lara [24]. In particular, the Folin–Ciocalteu assay was used for the assessment, wherein Na_2_CO_3_ (7.5% *w*/*v*) was used to neutralize the reaction. Following this, the sample was incubated at room temperature for 2 h. PHC were quantified in a microplate reader at 765 nm, with gallic acid used as a standard. The results were expressed as mg of gallic acid equivalents/100 g of dry weight (mg of GAE/100 g d.w.).

### 2.5. Assessment of ORAC

Antioxidant capacity of the energy snacks was determined by the method proposed by Bergvinson and García-Lara [25]. Briefly, the samples were diluted by 50 or 400-fold, by addition of phosphate buffer (pH 7.4), and extracts were obtained to separate soluble and bound phenolics. The resulting extracts were read in a microplate, wherein 25 μL of 2, 20-azobis (2-methylpropionamidine) dihydrochloride was injected as peroxyl radical generator, every 2 min for 1 h, prior to the fluorescence measurement. The resulting data was presented as μmol of Trolox equivalents per 100 g of dry weight (μmol TE/100 g d.w.).

### 2.6. Sensorial Evaluation

Samples for each formulation were tested for the acceptance of the product. Briefly, 50 non-trained panelists, aged 17–45 years and belonging to both sexes, were included as participants. Prior to the commencement of the test, all the participants were required to sign an agreement and confidentiality documents, which described the conditions of the assessment and acceptance of the utilization of the resulting data anonymously. For testing, individual spaces with adequate illumination were allotted at the Laboratory of Gastronomy, Universidad de Monterrey. The samples of the snacks were presented in a disposable tray at room temperature (25 °C). Each sample was randomly assigned a three-digit code, corresponding to four different samples, namely the control sample (no VM added), Provita C^®^, Provita C65^®^, and Probalance^®^. The order of the presentation was also assigned randomly. Prior to the evaluation and in-between the sample testing, the panelists were instructed to clean their mouths (tastebuds) with table water to remove any residual flavor. Besides this, the panelists were suggested to clean their sense of smell with coffee (contained in a glass bottle), by aspiring its aroma. A hedonic evaluation was structured with five points, scored on a scale varying from 0–5, where “0 = extremely dislike” and “5 = extremely like.” The characteristics (attributes) evaluated by the panelists included surface appearance, odor, flavor, consistency, and crunchiness [26,27].

### 2.7. Experimental Design and Statistical Analyses

The present study involved a totally randomized experiment. To assess the effect of processing factors, statistical analysis was performed using ANOVA, with confidence interval of 95%. Duncan’s Multiple Range Test was used (*p* ≤ 0.05) to assess the statistical differences between the means of various treatments. Statistical analyses were assessed using the SAS^®^ software, version 9.3 (SAS Institute Inc., Cary, NC, USA).

## 3. Results and Discussion

### 3.1. Nutritional Properties

**Figure 3:**
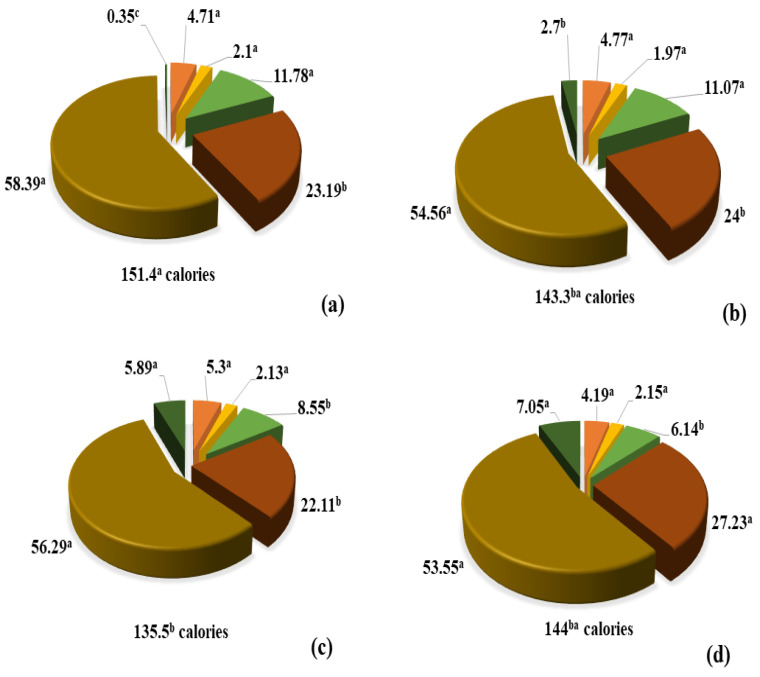
Chemical and nutritional analyses of the energy snacks, prepared with vegetable protein mixtures. (**a**) PC, (**b**) PC65^®^, (**c**) PB, and (**d**) control. Different letters among components are statistically different (*p* < 0.05). Total calories were calculated for a 30 g portion.

The nutritional components of the energy snacks are depicted in Figure 3. The moisture content of the samples remained unaffected by the addition of VM. For all samples, the moisture content was recorded to be in the range of 4.7–5.3%, which was in concordance with the values for a similar snack formulation reported by Caipo et al. [28]. Additionally, the results for the ANOVA analyses (*p* = 0.7060) also showed that there were no statistically significant differences among the four formulations of snacks. The low moisture content of the snacks could be attributed to relatively high temperatures and interaction of the starch, present in the pseudo-cereals, with the lipids present in the cacao liquor and the coconut milk [29]. In general, lipids are hydrophobic in nature, and thus cannot interact with water owing to lack of formation of dipoles. The presence of lipids does not allow the adsorption of relative humidity from the environment by the snacks [30].

As expected, the primary macronutrient present in the developed formulations was carbohydrates, which was measured to be in the range of 53–58%. Results for the ANOVA analyses showed that the addition of VM conferred no significant effect (*p* = 0.1285) on this parameter. Total carbohydrates recorded in the formulations, developed in the present study, were lower as compared to the energy bar developed by Caipo et al. [28], which comprised of quinoa, amaranth, and cañihua (*Chenopodium pallidicaule*) (>64%). Interestingly, these values were similar to those reported for a nutritive cereal bar that contained egg albumin and milk powder (57%) [31].

The complex carbohydrates present in foods, such as starch, are considered to be beneficial for human health, especially for sedentary populations that are at risk of chronic diseases [32]. Quinoa contains starch comprising of units of D-xylose, D-ribose, D-galactose, and maltose, which have a low glycemic index. However, it also contributes to a high glycemic response, and can be considered as an energy product owing to the presence of saccharose and fructose components [33]. This is concerning with regard to the puffing process of quinoa, as it can modify the chemical structure of starch and improve its digestibility via gelatinization of starch and degradation of dietary fibers [34,35].

For the content of total and reduced sugars, the ANOVA analyses showed that the addition of VM did not have any significant effect on these two parameters (*p* = 0.2259 and *p* = 0.5275, respectively). The analysis of these parameters is particularly essential as its consumption is associated with a rise/reduction in the blood glucose levels. Total sugars were recorded to be ~11.8% and ~13.6% for PB sample and PC65 snacks, respectively. The last treatment probably resulted in the highest value as this protein concentrate contained ~40% of carbohydrates, as per the technical and nutritional specifications [19]. The amount of total sugars was recorded to be in the range of 8.6% for PC65 and 10.4% for the control samples. In general, simple sugars (monosaccharides) are positively correlated to hyperglycemia owing to their rapid absorption [36]. All energy snacks produced in this study showed significantly lower amounts of total and reduced sugars as compared to the commercial cereal-based meals, which contain up to 30 g per 100 g of product [37].

For protein content, the ANOVA analyses showed that the addition of VM incurred a high and statistically significant effect (*p* = 0.0006). In particular, the samples PC and PC65 presented the highest amounts of this macronutrient as compared to PB and the control. It was inferred that the samples of the snacks showing highest values of protein were those that were elaborated with VM containing more crude proteins. For instance, Provita C^®^ and Provita C65^®^ contained 88% and 60%, respectively, of this compound in their original formulas. This was followed by Probalance^®^, which contained 20% of this compound [19]. This is in agreement with the results obtained in the present study, and corroborated that the addition of this type of formulations into the bars can be used a promising alternative to increase the protein content for consumers with special nutritional needs. The protein content present in the energy snack samples, reported in the present study, was similar to the products produced using animal protein sources or similar products, which were aimed at increasing the nutritional quality [38]. Nevertheless, the amino acid profile of quinoa makes them an excellent alternative to improve the nutritional value of such kinds of products. Also, the protein contents recorded in the developed energy snacks were higher as compared to the cereal (mixture of rice and oat flakes) snack reported by Srebernich et al. [39], which comprised of acacia gum, inulin, and sorbitol, with protein content in the range of 3.8–3.9%. The Food and Agriculture Organization [40] has recommended the consumption of 1 g/kg/day of protein to fulfill the daily requirements of this nutrient in adults (aged > 8 years). Thus, the consumption of 30 g of the developed product will provide 3 g of proteins, resulting in the fulfillment of 6% of the daily protein requirement for an adult of 50 kg.

The ANOVA analyses showed that the content of lipid content was also significantly affected by the addition of VM in the snacks (*p* = 0.0444). As expected, the control bar was characterized by the highest amount of lipids, and no statistically significant differences were recorded among the other snacks’ samples upon VM addition. All samples presented more elevated amounts of fats as compared to other products. However, these macronutrients were majorly contributed by cacao liquor, quinoa seeds, and coconut milk. In particular, the lipid content in the energy snacks comprised of monounsaturated (C15:1, C16:1, C17:1, C18:1, and C:20:1), polyunsaturated (C18:2 and C18:3), and small amounts of saturated fatty acids (C12:0, C14:0, C15:0, C16:0, C17:0, C18:0, C20:0, and C22:0). The ingestion of unsaturated fatty acids has been previously shown to affect cardiovascular health, via reduction of cholesterol and low-density lipoproteins in the blood [41].

Despite the high amounts of unsaturated fatty acids, the ratio of ω-6/ω-3 is important for further analysis, particularly to measure the contribution toward the potential benefits, such as antioxidant activity [5]. The contribution of lipids to the diet should be able to achieve 30–35% of the total calories intake per day. The consumption of 30 g of a PC snack provided 7 g of lipids (63 calories). Based on a 2000 calorie diet advised for an adult, it constituted 10.5% of the recommended fats intake (600 calories, 30%) [42]. 

The results for the ANOVA analysis showed that total dietary fiber content was significantly affected by the addition of VM (*p* = 0.0002). Similarly, multiple mean comparison analysis demonstrated that the control and PB snacks were characterized by the highest values for the total dietary fiber content. In particular, some of the ingredients, namely quinoa, amaranth [43], and coconut milk are known to contain high amounts of dietary fiber, which contributed to the observed high values in the control snack. On the other hand, PB snack showed high dietary fiber content when compared with PC and PC65. This could be attributed to the presence of ~35% of dietary fiber in PB vegetal protein mixture, according to the datasheet [19]. Considering the importance and benefits of total dietary fiber in health, its daily consumption must reach 25 g/per day, based on a 2000 calorie diet [44]. Thus, consumption of 30 g of a PB snack provides 1.8 g of dietary fiber, combined with other sources of these nutrients that further assist in proper functionality of the gastrointestinal system.

For the assessment of inorganic material (ash content), the ANOVA analyses showed that the addition of VM did not show any significant effect on the total trace elements content (*p* = 0.7255). Thus, no differences were recorded among the snack formulations for this parameter. However, values of inorganic material were recorded in the range of 1.9–2.1%, and the highest value was observed for the control snack. This could be attributed to the presence of high amounts of trace elements in pseudo-cereals as well as coconut milk and cocoa liquor, which are considered to be a good source of phosphorous, magnesium, potassium, calcium, zinc, and iron [45,46]. Despite the fact that the snacks were prepared only with vegetal foods, inorganic material content was higher as compared to other products prepared using animal food ingredients and additives, such as those reported by Sant’Ana et al. [38]. Although the developed snacks did not highlight exclusively from different formulations in terms of their inorganic contents, these snacks could be considered as a good source of trace elements, and thus could be used as an alternative for vegetarian and vegan consumers.

In terms of total caloric values per 30 g portion of the snack (Figure 3), the ANOVA analyses showed that the addition of VM conferred significant effect on this parameter (*p* = 0.0224). Particularly, PC snack was characterized by the highest caloric content. However, the control and PC65 samples could be considered statistically equal. Interestingly, PB snacks displayed the lowest (*p* < 0.05) caloric content when compared with other samples. In a previous study, Green et al. [45] analyzed a total of 171 snack foods, and sorted them into several types (including “healthy” and “unhealthy”), such as formulated bars, corn-based bars, granola bars, and others. The nutritional quality and caloric values (energy content) of these snacks were reported in the range of 76–214 calories per portion. The snacks described in the present study provided caloric content similar to the products offered in the market; however, the nature of the constituting ingredients ensured a highly added nutritional value. Some of the previous studies have established that whole foods, high in protein and fiber, and whole grains enhanced satiety when consumed as a snack [46]. For the energy snacks, developed in the present study, calories per portion were slightly higher as compared to the nutritive cereal bars reported by Sant’Ana et al. [38], produced using egg white, milk powder, honey, soybean, and sucrose (121.2 calories per portion).

Lastly, the consumption of any energy snack developed in this study would provide a calorie intake of 135.5–151.4 calories per 30 g portion, which is equivalent to 6.7–7.5% of calories based on a 2000 calorie diet. However, the snacks could be regarded as hypercaloric product, considering the small amount of portion (30 g). Thus, further analysis should be considered to reduce the caloric content of the developed samples.

### 3.2. Contents of Total, Free, and Bound PHC in the Energy Snacks

Unfortunately, no information is available regarding the daily dosage of specific antioxidants compounds, such as PHC [47]. However, regular intake of antioxidants is known to play an essential role in reducing the risk of certain non-communicable diseases [48,49]. The ANOVA analysis showed that the addition of different VM to the snacks conferred a significantly high impact on the contents of free (*p* < 0.0001) and bound PHC (*p* < 0.0001).

**Figure 4:**
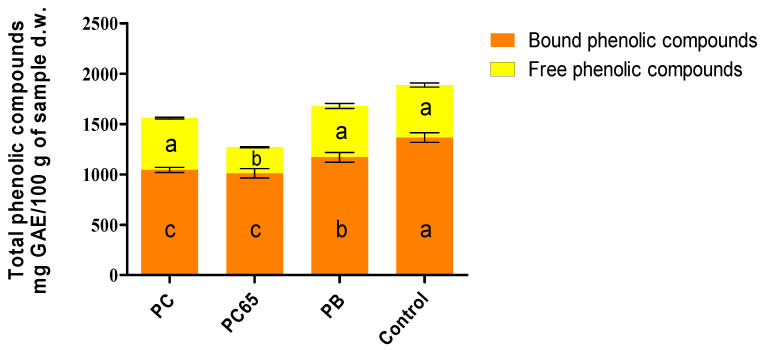
Contents of total, free, and bound phenolic compounds in the energy snacks. Different letters present in the same column color are statistically different (*p* < 0.05).

Figure 4 depicts total, free, and bound PHC present in the energy snacks. For the control snack, higher content of bound PHC (1366 mg of GAE/100 g d.w.) was recorded, which could be attributed to higher content of cacao liquor, coconut milk, and oil, when compared with other formulations [12,50]. PB snack contained 1169.3 mg of GAE/100 g d.w., whereas PC65 and PC snacks showed the lowest values for this parameter (*p* < 0.05), with 1010.4 and 1044.1 mg of GAE/100 g d.w., respectively. The highest values for free phenolic acid content were recorded in the control (520.7 mg of GAE/100 g d.w.), PB (508.6 mg of GAE/100 g d.w.), and PC (514.5 mg of GAE/100 g d.w.) snacks. PC65 snack showed a significantly lower value (258.5 mg of GAE/100 g d.w.) when compared with other samples. These effects could be contributed by interaction between proteins and free PHC. Energy bars prepared with Probalance^®^ (containing 20% of proteins) and the control sample showed the lowest concentrations for protein content (Figure 3). The latter could have contributed to the observed improvement in the quantification of free phenol molecules, as the aforementioned interactions between proteins and free phenol would not have occurred in the samples with comparatively lower protein contents.

To accomplish a discussion and effectively compare the data with previous studies, the total phenolic acid content was calculated in terms of the sum of free and bound compounds. Nevertheless, it is important to consider that other type of compounds or molecules could have reacted during the analysis, which might have affected the results [51,52] [Melini 2021, Carciochi 2016]. In such a case, even Folin–Ciocalteu assay could be a limiting factor in the present study. Thus, the present data was compared with studies that utilized the same method for the quantification of PHC in similar samples. However, the double extraction process used for these compounds could have also resulted in a significant increase in their respective concentration. In this regard, the control, PC, PB, and PC65 contained 1886.7, 1558.6, 1677.9, and 1268.9 mg of GAE/100 g d.w., respectively. In comparison to this, the results for some of the snack samples (the control and PB) were found to be higher as compared to the cereal bars reported by Marques et al. [53], which were prepare with flours of acerola residues, enriched with antioxidant substances and fiber,. For these cereal bars, the content of phenolic compounds was recorded in the range 330–1600 mg of GAE/100 g d.w. of the product. However, these formulations also utilized brown sugar as an ingredient, which was not desirable. A similar tendency was recorded for values of total phenolic compounds for vegetable-enriched corn-based extruded snacks reported by Bisharat et al. [54]. In particular, total phenolic contents were recorded in the range of 15–25 mg of GAE/100 g d.w. for broccoli enriched extrudates and 30–50 mg of GAE/100 g d.w. for olive paste enriched extrudates.

Similarly, Silva Carvalho and Conti Silva [55] evaluated the total phenolic compounds for cereal bars formulated with banana peel flour, and reported values between 87–419 mg GAE/100 g for seven different formulations, which contained variable amounts of rice flakes, oat flour, and banana peel flour. Ahmed and Abozed [56] developed a functional and novel snack enriched with *Hibiscus sabdariffa* by-product, with the aim to increase antioxidant activity and phenolic contents. The study reported total phenolic contents in the range of 599–1757 mg GAE/100 g. The results for the present study were considerably higher, and this attributed to the nature of the main ingredients used in the energy snacks. It has been previously reported that cocoa powder is rich in polyphenols, such as (+)-catechin, (–)-epicatechin, oligomers of these monomeric base units, namely procyanidins, and anthocyanidins. It is also known to contain monomers to tetradecamers of other similar compounds [57]. Besides this, indigenous grains usually contain high amounts of flavonoids and other phenolic compounds. For instance, quinoa grains have been shown to contain caffeic acid, ferulic acid, p-coumaric acid, p-OH-benzoic acid, and vanillic acid [10]. In addition to the components found in quinoa, amaranth seeds also contain synaptic acid, protocatechuic acid, and some betacyanins (amaranthine, iso-amaranthine, and betanin) [9]. Additionally, the extraction method could have possibly influenced the concentration values of phenolic compounds in the snacks developed in the present study.

In addition to this, it is important to assess the interaction of phenolic compounds with vegetal protein fractions [58]. Such reactions could possibly occur during food processing, and the formation of complexes between phenolic compounds (ferulic acid, catechin, and similar compounds) and protein fractions present in cereals and pseudocereals (albumins, globulins, prolamins, and others) might be observed [59]. Such interactions could in turn affect the functionality, bioavailability, and physiological activity of the products [60]. Some of the inhibitory effects of phenolic compounds on the digestive process of food with high energy density ingredients, like carbohydrates and lipids, are considered to be beneficial if consumed in weight-controlled regimes. However, the inhibition of protein digestion is not desirable as it would result in a reduction in the bioavailability of amino acids, which would further affect the nutritional status of the consumers [61]. 

### 3.3. ORAC Analyses in the Energy Snacks

**Figure 5:**
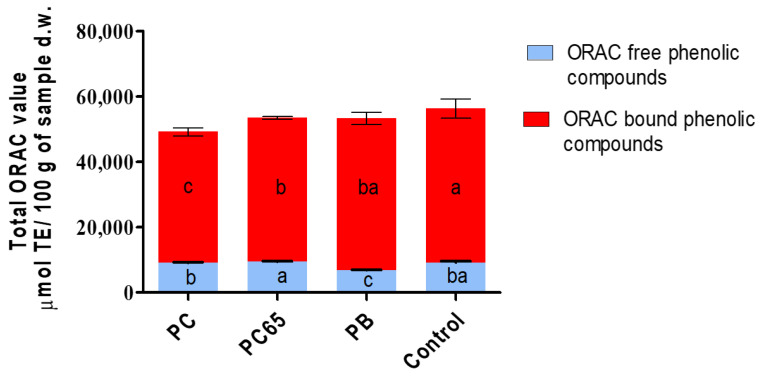
ORAC values for the contents of total, free, and bound phenolic compounds present in the energy snacks. Different letters present in the same column color are statistically different (*p* < 0.05).

Figure 5 depicts the results of ORAC analyses for free and bound phenolic compounds present in the developed snacks. Since the values for the content of bound phenolic compounds were higher (Figure 4), the ORAC values were also recorded to be higher as compared to free PHC, that too in a directly proportional manner. The ANOVA results showed that the addition of VM had a significantly high impact on ORAC for both free (*p* = 0.0001) and bound (*p* = 0.0009) phenolic compounds of the samples.

For free phenolic compounds, PC65 snack showed highest ORAC value of 9392.7 μmol TE/100 g d.w., whereas the lowest value was recorded for PB snack, with ORAC value of 6769.9 μmol TE/100 g d.w. No statistically significant differences (*p* < 0.05) were recorded for the ORAC values of the control snack when compared with PC and PC65 snacks. For bound phenolic compounds, PC65 and the control snacks showed the highest (*p* < 0.05) ORAC values of 47,087 and 46,531 μmol TE/100 g d.w., respectively. In comparison to these, PC snack sample showed the lowest ORAC value (40,158 μmol TE/100 g d.w.) for bound phenolic compounds.

To accomplish a discussion and compare the obtained data with other studies, the total ORAC values for the snacks were calculated in terms of the sum of ORAC for free and bound phenolic compounds. Interestingly, the control snack accounted for the highest capacity with 56,279.3 μmol TE/100 g d.w; followed by PC65 snack with 56,479.7 μmol TE/100 g d.w., PB snack with 53,293.9 μmol TE/100 g d.w., and PC snack with 49,192.9 μmol TE/100 g d.w. Indirectly, it could be inferred that protein–PHC interactions could have affected the values for antioxidant capacity [58], especially for the bars containing highest amounts of protein (Figure 3), which resulted in lower ORAC values for PC samples produced using VM with minimum protein concentration. Thus, higher the protein content, lower would be the content of bound PHC and ORAC values.

Antioxidant activity of the produced snacks was found to be very different from the cereal bars reported by Rosales et al. [62], produced with Merlot/Cabernet Sauvignon grape seed flour, clover honey, oat flakes, pure cane sugar, vegetable oil, and cinnamon. The study reported antioxidant activity of 513–1103 μmol TE/100 g d.w. for cereal bars produced with Merlot grape seed flour as an antioxidant powder. These values were extremely lower as compared to the energy snacks developed in the present study, with exception of the control snack that corresponded to 1.9% of the antioxidant activity. Silva Carvalho and Conti-Silva [55] reported antioxidant activity values in a range of 60–341 μmol TE/100 g d.w. for cereal bars formulated with banana peel four, evaluated using the ABTS + method. These values were extremely inferior when compared with the snacks developed in the present case. A similar trend was reported by Laokuldilok et al. [63], wherein encapsulated turmeric extracts were added to extruded cereal snacks to increase their antioxidant activity. Total phenolic compounds were very low (662–207 μmol TE/100 g) as compared to those reported in the present study.

The occurrence of extremely high values for both the quantification of phenolic acid compounds and ORAC determinations could be attributed to the enhanced separation during the extraction of free and bound phenolic acid compounds in the samples. Thus, higher radical scavenging activity was observed during the assays. The quantification of relative content of bioactive compounds and assessment of interactions between these molecules and other food matrix nutrients represent the main steps that are required for the determination of total antioxidant capacity and, consequently, evaluation of potential health benefits [64].

### 3.4. Sensory Attributes

**Figure 6:**
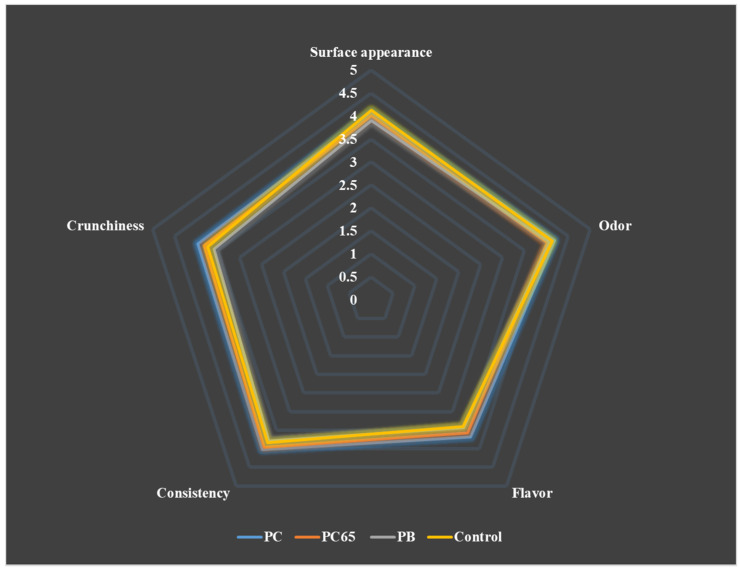
Evaluation of the sensory attributes of the snack bars. Here, 1 = “dislike extremely”, 2 = “dislike slightly”, 3 = “neither like nor dislike”, 4 = “like slightly”, and 5 = “like extremely”.

For the evaluation of sensory attributes, 50 panelists (aged 17–45 years) belonging to both genders (66% women and 34% men) were recruited. The results for the assessment of sensory attributes are presented in Figure 6.

No statistically significant differences were recorded among sensory attributes of four formulations (*p* > 0.05); however, flavor and crunchiness showed highest values for standard deviation. For four samples that were evaluated, average values were recorded between 3.4 and 4.1, which corresponded to “neither like nor dislike” and “like slightly,” respectively. The trend showed that PC snack was highly accepted by the panelists, with an overall average value of 3.95. None of the samples received the maximum scale punctuation (5, “like extremely”). This suggested that formulations must be improved further to increase the score in sensory analysis, even though all the snacks could be considered as an acceptable meal option as per current scoring.

## 4. Conclusions

The present study achieved the development of snacks with an acceptable nutrient quality, which were prepared using ancestral pseudo-cereals, vegetal mixtures, and lipid sources. An easy manufacturing method was utilized for the development of energy snacks. The addition of VM showed a statistically high impact on protein, lipids, total dietary fiber contents, and total calories per portion. However, such types of foods should not be consumed daily or frequently. Additionally, VM showed a significant effect on the contents of free and bound PHC and the antioxidant activity, especially in the control and PC65 samples. This could possibly act as a limiting factor when compared with similar samples. Evaluation of sensory attributes demonstrated high acceptance by the panelists for all the developed formulations, as no differences were detected among the samples. Further analyses are required to corroborate the antioxidant effects and beneficial effects (digestibility) of the control and PC65 snacks on health via in vitro and in vivo analyses. The results for such kind of studies would aid in better understanding and development of different food formulations that include non-conventional ingredients and less processed foods for consumers.
